# Post-operative events following elective craniotomy for tumor in children

**DOI:** 10.1007/s11060-025-05239-y

**Published:** 2025-10-20

**Authors:** C. Stewart Nichols, Emal Lesha, Delaney Graham, David G. Laird, Brandy Vaughn, Nir Shimony, Paul Klimo Jr.

**Affiliations:** 1 College of Medicine, University of Tennessee Health Science Center, 910 Madison Avenue, Memphis, TN 38163 USA; 2 Department of Neurosurgery, University of Tennessee Health Science Center, Memphis, TN USA; 3 Neuroscience Institute, Le Bonheur Children’s Hospital, Memphis, TN USA; 4 Semmes Murphey, Memphis, TN USA; 5 St. Jude Children’s Research Hospital, Memphis, TN USA

**Keywords:** Brain tumor, Postoperative event, Complication, Predictors, Elective, Craniotomy, Pediatric

## Abstract

**Introduction:**

Craniotomy for tumor resection in the pediatric population can result in many potential postoperative events (POEs). POEs, in turn, are a function of many variables, such as tumor type, size, location, surgeon experience and goal(s) of surgery. The current literature is limited to either specific POEs or complications associated with specific tumor pathologies.

**Objectives:**

The primary purpose of this study was to undertake a holistic approach and identify those variables predictive of all types of POEs and only surgical ones, following elective craniotomy for tumor in a diverse pediatric population.

**Methods:**

All elective craniotomies for tumor resection performed from 2010 to 2022 were included, excluding patients > 21 years of age. Demographic, clinical, and procedural covariates for each encounter (i.e., operation) were collected. POE was defined as a postoperative incident (medical or surgical, expected or unexpected) that necessitated further diagnostic testing, evaluation, or intervention within 90 days of surgery. Bivariate and multivariate analysis were performed; backward model selection was done to yield a final multivariate model retaining only variables with *p* < 0.05. Adjusted odds ratios (ORs) and 95% confidence intervals (CIs) were reported.

**Results:**

A total of 1,276 patients underwent 1,497 elective craniotomies for tumor resection with a median age of 9.45 years at index operation. In 535 (36%) encounters, there was at least one POE: 329 (61%) had surgical only, 109 (20%) had medical only, and 97 (18%) had both surgical and medical POEs. Multivariate analysis found several predictors of all types of POEs: length of stay (LOS) > 7 days, length of ICU stay (ICUS), attending surgeon, surgical time, and prior craniotomy. All of these, in addition to previously treated hydrocephalus and tumor type, were predictors of surgical-only POEs. All identified covariates were promoters of POE except for tumor type and redo craniotomy, which were protective.

**Conclusion:**

Analysis of our large pediatric tumor database identified several key statistical drivers of all POEs and surgical-only POEs following elective tumor resection, of which LOS > 7 days was the strongest.

**Supplementary Information:**

The online version contains supplementary material available at 10.1007/s11060-025-05239-y.

## Introduction

In the United States, tumors of the central nervous system are the most common cancer in children aged 0–19, as well as the leading cause of cancer-related death in children 0-14 [1]. Despite an ever-increasing number of therapeutic drug options, surgery remains a critical part in the treatment of these children. Each craniotomy comes with its own risk profile, with short-term morbidity ranging from 24% to 44% [2–5] and persistent and/or permanent morbidity ranging from 8% to 19% [2, 3, 5]. Postoperative events (POEs)—whether neurologic or systemic—can negatively affect functional status and quality of life in these patients [6].

Most prior studies concerned with surgical morbidity in pediatric neurosurgery are ones that broadly examine all neurosurgical procedures [2, 5, 7, 8] or a single procedure with multiple indications [6, 9]. Conversely, the literature that does pertain to craniotomies for tumor is often quite specific, focusing on a particular tumor pathology [10–13] or postoperative complication [14–16].

From our database of elective craniotomies for tumor spanning a 12-year period, we’ve demonstrated that POEs play a major role in increasing length of stay (LOS) beyond seven days [17]. This study seeks to build upon those findings by analyzing relationships between patient/surgery characteristics and the occurrence of both surgical and medical POEs.

## Methods

These methods were previously published by the authors [18] as quoted below:

“A departmental database was created and maintained by a sole research coordinator (B.V.) in which all craniotomies performed for tumor resection at the tertiary care children’s hospital were recorded prospectively for clinical research. Data analysis was completed retrospectively after the conclusion of the study period. All patients older than the age of 21 were excluded from data collection as that is the age cut-off that our hospital designates one as being an adult. All qualifying procedures from the period of Jan 1, 2010, to Dec 31, 2022, were included: elective (i.e., not urgent or emergent) craniotomy (de novo or repeat) for any degree of resection of tumor. All other cranial procedures for the treatment or diagnosis of a tumor (e.g., open biopsy, endonasal surgery, laser ablation) were excluded.

“Demographic, clinical, and procedural variables were recorded (Tables 1 & 2). These included but were not limited to race, age, sex, shunt presence, primary surgeon, location and type of tumor, presence of cancer predisposition syndrome, and the occurrence of one or more medical or surgical postoperative event (POE). Age was analyzed as both a continuous variable and categorical (see additional details below): infant/toddler (0–4 years), young child (5–9 years) and pre-teen/teen/young adult (10 years and older). There were 3 surgeons (A-C) during the study period, but the vast number (94%) of the operations were performed by surgeons A and B.” Surgeon A was 15 years senior to Surgeon B and thus had more experience than Surgeon B throughout the duration of data collection; both dedicated a large portion of their practice to neuro-oncology. All surgeons in the study were fellowship-trained in pediatric neurosurgery.

“A patient with preexisting treated hydrocephalus was one who was dependent on a shunt or a patent third ventriculostomy at the time of their resection. Prior craniotomy was either “no” or “yes” - either at the same site or different. All tumor types were recorded, but for statistical purposes tumor grade was collapsed into either high grade or low based on histologic and molecular analysis. The few intermediate grade tumors or those that could not be graded were classified as high and low, respectively.” [18] (For tumor type, “Other” included any tumor that did not fall under the major categories listed in Tables 2, 3 and 4, such as choroid plexus papilloma or meningioma).

“Returning to the neurosurgical OR due to POE was a variable that occurred during the index admission—after the index elective craniotomy for tumor and before discharge. Readmission was defined as readmission to the hospital for any reason and to any service within 90 days after the date of discharge from the index admission for the elective tumor operation. If multiple craniotomies for tumor resection were conducted during a single hospital stay, then only the last craniotomy was included for analysis with the date of the last index operation used as a surrogate for hospital admission date.” [18].

### POE definition and classification

A POE was a postoperative incident (medical or surgical, expected or unexpected) that necessitated further diagnostic testing, evaluation, or intervention within 90 days of the index operation. This time period was meant to capture POEs that occurred during the index admission as well as during any readmission. We felt that the term “POE” was a more accurate description of the outcomes we were studying rather than the word “complication”. Classifying a POE as expected versus unexpected was made independently by the research coordinator, and based on information such as tumor location, goal(s) of surgery, review of surgeon’s notes detailing risk analysis, and other pertinent information. Minor or transient post-operative physical exam or laboratory abnormalities that did not require additional intervention or that resolved or were resolving without additional intervention at time of discharge were not considered POEs. Our goal was to capture POEs that any reasonable practitioner would consider to be significant.

Preoperative findings that remained stable after surgery, such as a history of diabetes insipidus (DI) or known extremity weakness, were not considered POEs. For patients who underwent more than one elective craniotomy for tumor in a single hospital admission, only new or worsening POEs following the last surgery were considered for analysis (i.e., a new deficit following the first surgery that remained stable after the second surgery would not be considered a POE, as it functions similar to other preoperative findings that did not worsen with surgery).

Surgical POEs included the following categories: expected new neurologic deficit, unexpected new neurologic deficit (improving), unexpected new neurologic deficit (not improving), seizure, bacterial meningitis, aseptic meningitis, wound infection, brain abscess, hemorrhage (managed medically), hemorrhage (return to OR), cerebrospinal fluid (CSF) leak, pseudomeningocele, hydrocephalus/hygroma, arterial stroke, venous stroke, venous sinus thrombosis, and other. Examples of “other” surgical POEs were cerebral edema, uncontrolled pain, pin site skull fracture, hallucinations, and wound dehiscence.

Medical POEs were categorized as cardiac, respiratory–atelectasis was only included if treatment was required—gastrointestinal/hepatic, renal, hematologic (including thromboembolism), new onset DI, hyponatremia (Na < 130) or hypernatremia (Na > 150), and other. “Other” medical POEs included findings such as septicemia, sacral ulcer, central hypothyroidism, and viral infection.

### Statistical analysis

Statistical data analysis was done using SPSS Version 29 (Armonk, NY: IBM Corp.). Categorical data was summarized by frequency and percentages, and continuous variables were reported using medians (Mdn) and interquartile ranges (IQR). Bivariate and multivariate analysis were performed using generalized estimating equations (exchangeable correlation structure) to address patients with multiple hospitalizations and the longitudinal nature of data. Backward model selection was done to yield a final multivariate model retaining only variables with *p* < 0.05. Adjusted odds ratios (ORs) and 95% confidence intervals (CIs) were calculated based on the above analyses.

## Results

**Fig. 1:**
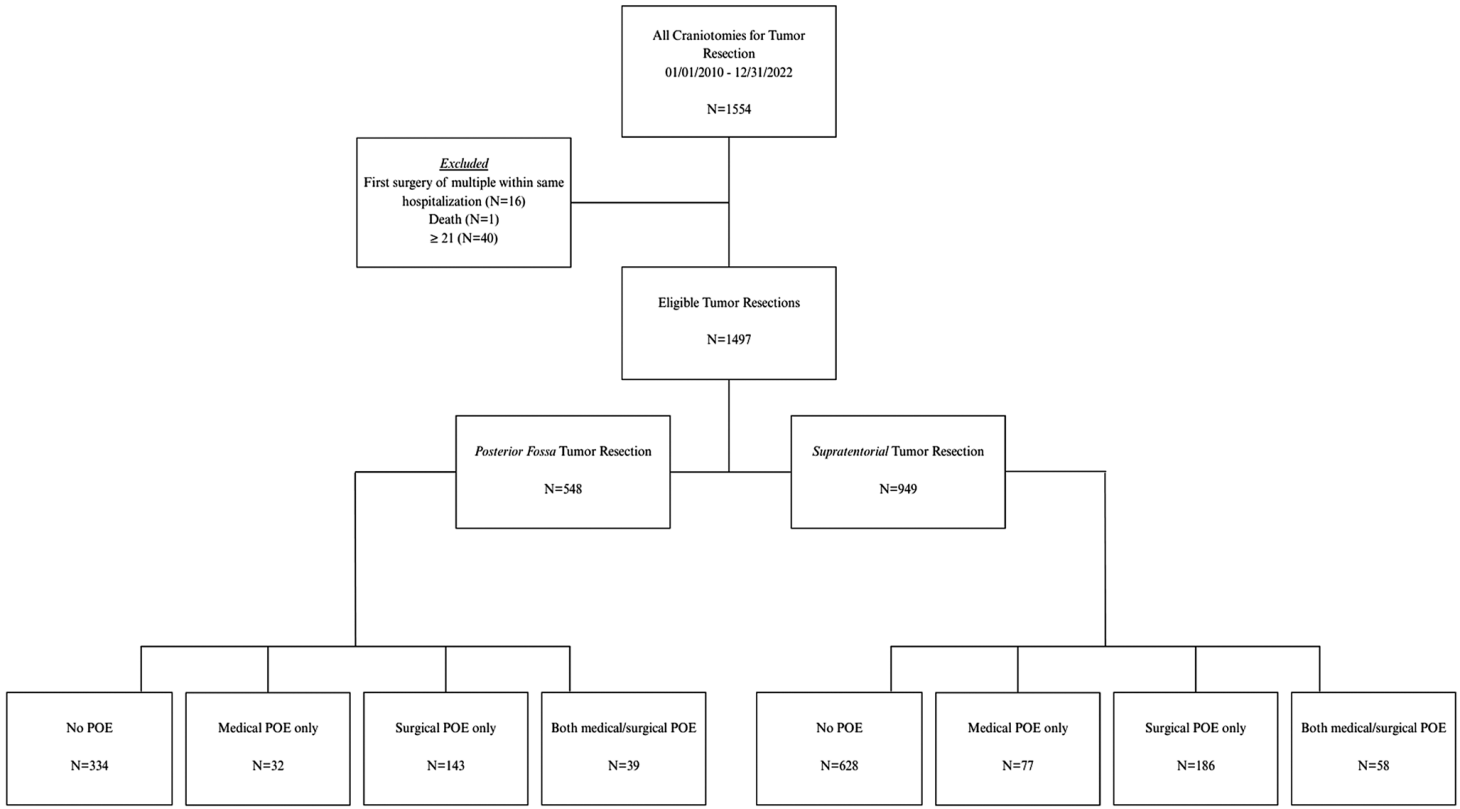
Study eligibility, inclusion criteria, and case distribution

There were *n* = 1554 operations that met the criteria for tumor resection craniotomies. Sixteen (*n* = 16) operations were excluded as the first surgery during a hospital visit requiring two (*n* = 2) elective craniotomies. Finally, we excluded patients older than 21 years (*n* = 40) and one (*n* = 1) patient who died due to complications secondary to pneumonia more than 2 weeks after surgery. Ultimately, 1497 total encounters were included for analysis (Fig. 1). Table 1 describes our patient population as previously published [17, (p202)]. There were 17 *n* = 1276 unique patients; 17% (*n* = 221) had two encounters for elective craniotomy for tumor. Most craniotomies for tumor were supratentorial (63.3%), and the median age at surgery was 9.45 years (IQR 4.43–14.1. The majority had a LOS less than or equal to seven (7) days (88.1%) and had no history of endoscopic 3rd ventriculostomy (ETV, 93.7%) or shunt (85.0%) or cancer predisposition syndrome (94.9%). Most encounters were without any POE (64.3%); 7.3% resulted in only medical POEs, 22% only surgical POEs, and 6.5% both medical and surgical POEs. Fifty-one (51, 3.4%) patients returned to the neurosurgical OR due to a POE during the index admission. Characteristics of the encounters based on the occurrence of surgical POEs or any POEs are demonstrated in Tables 2 and 3, respectively. New, expected neurological deficits occurred in 188 (12.6%) encounters and unexpected neurological deficits in 133 (8.9%) encounters, as demonstrated in Online Resource 1. The most common surgical POE was new but expected neurological deficit; the most common medical POE was hypo- or hypernatremia (*n* = 97, 6.5%) (Online Resource 2).

**Table 1:**
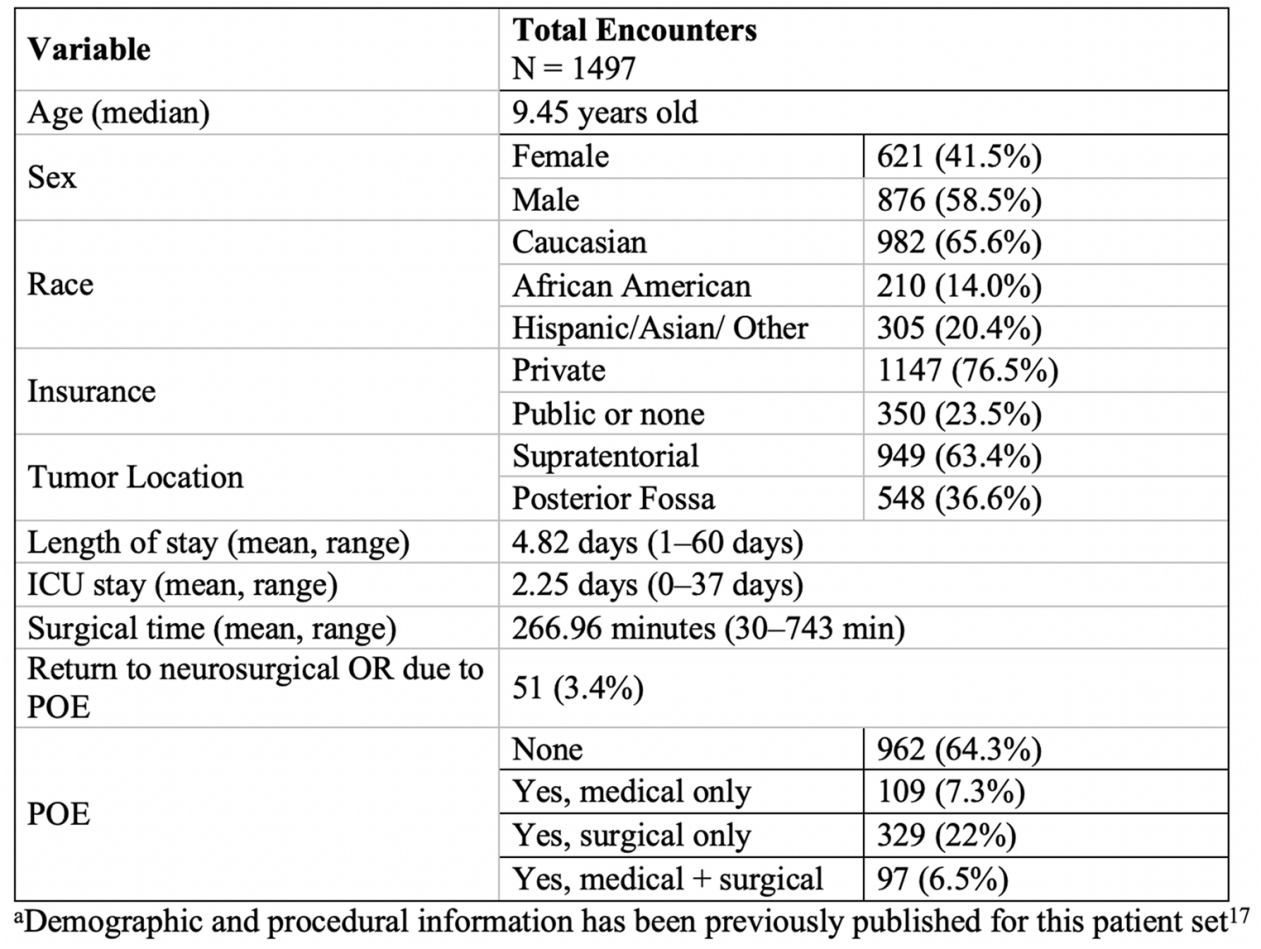
Encounter characteristics and patient demographics^a^

**Table 2:**
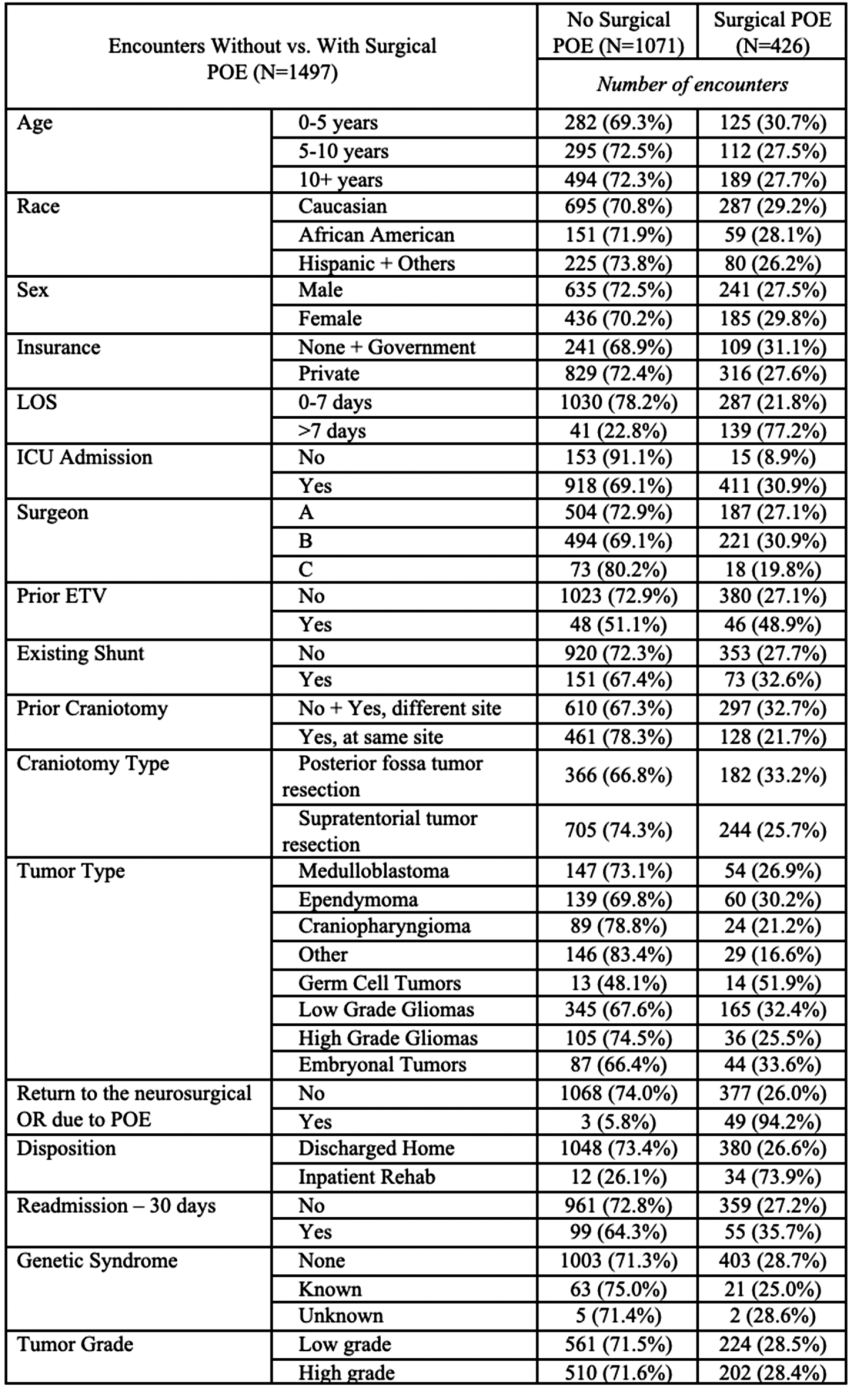
Encounter characteristics between patients without surgical POE and those with surgical POE

**Table 3:**
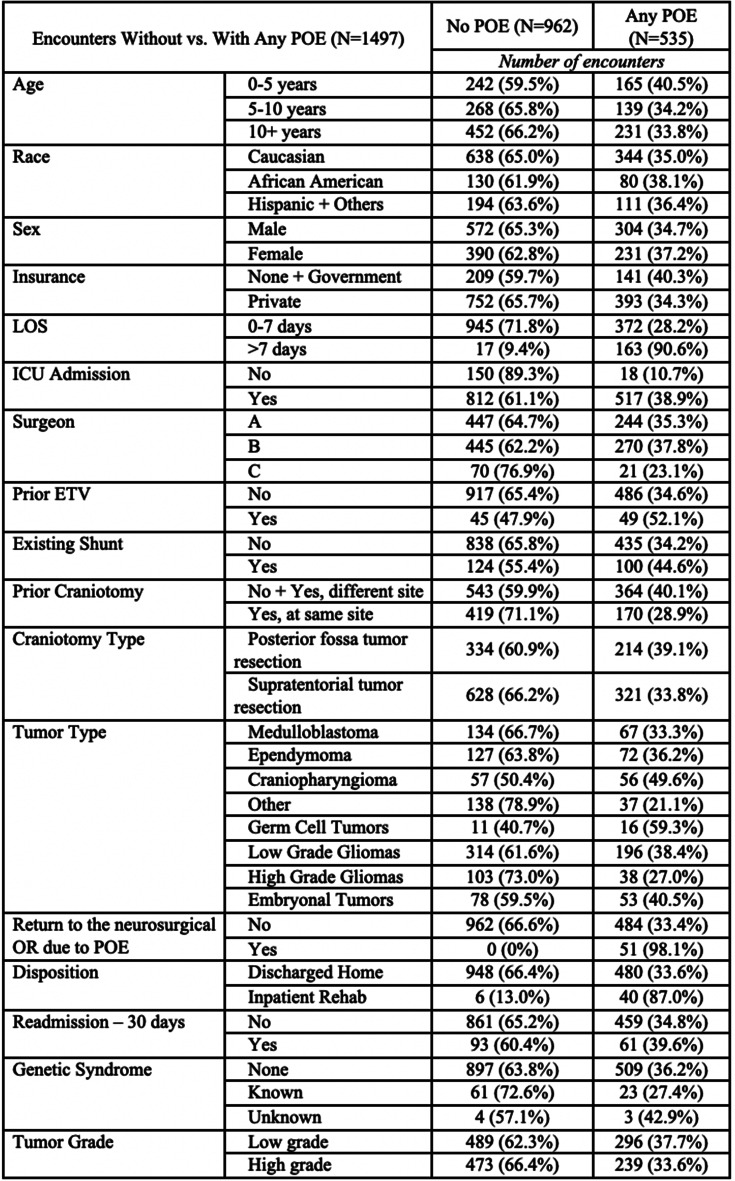
Encounter characteristics between patients with no POE and those with any POE

### Surgical POE

Bivariate analysis of surgical POE occurrence revealed several significant variables (Online Resource 3): continuous LOS (OR 1.51, *p* < 0.001), LOS > 7 days (OR 3.3812.17, *p* < 0.001), ICU admission (OR 4.57, *p* < 0.001), length of ICU stay (ICUS) (OR 1.48, *p* < 0.001), surgical time (OR 1.006, *p* < 0.001), surgeon B (OR 1.81. *p* = 0.03), prior ETV or shunt (OR 2.58, *p* < 0.001), and germ cell tumor (OR 2.25, *p* = 0.04). There were other significant variables with ORs less than one, including prior craniotomy at the same site (OR 0.57, *p* < 0.001), supratentorial craniotomy (OR 0.70, *p* = 0.002), craniopharyngioma (OR 0.56, *p* = 0.02), and other tumor type (OR 0.42, *p* < 0.001). Multivariate analysis showed the following to be significant predictors of surgical POE (Table 4): LOS > 7 days (OR 3.38, *p* < 0.001), ICUS (OR 1.24, *p* < 0.001), surgeon B (OR 2.92, *p* = 0.004), surgical time (OR 1.004, *p* < 0.001), and prior ETV or shunt (OR 1.82, *p* = 0.02); conversely, prior craniotomy at the same site (OR 0.57, *p* < 0.001), craniopharyngioma (OR 0.33, *p* < 0.001), and other tumor type (OR 0.40, *p* = 0.001) were protective or less likely to result in surgical POE.

**Table 4:**
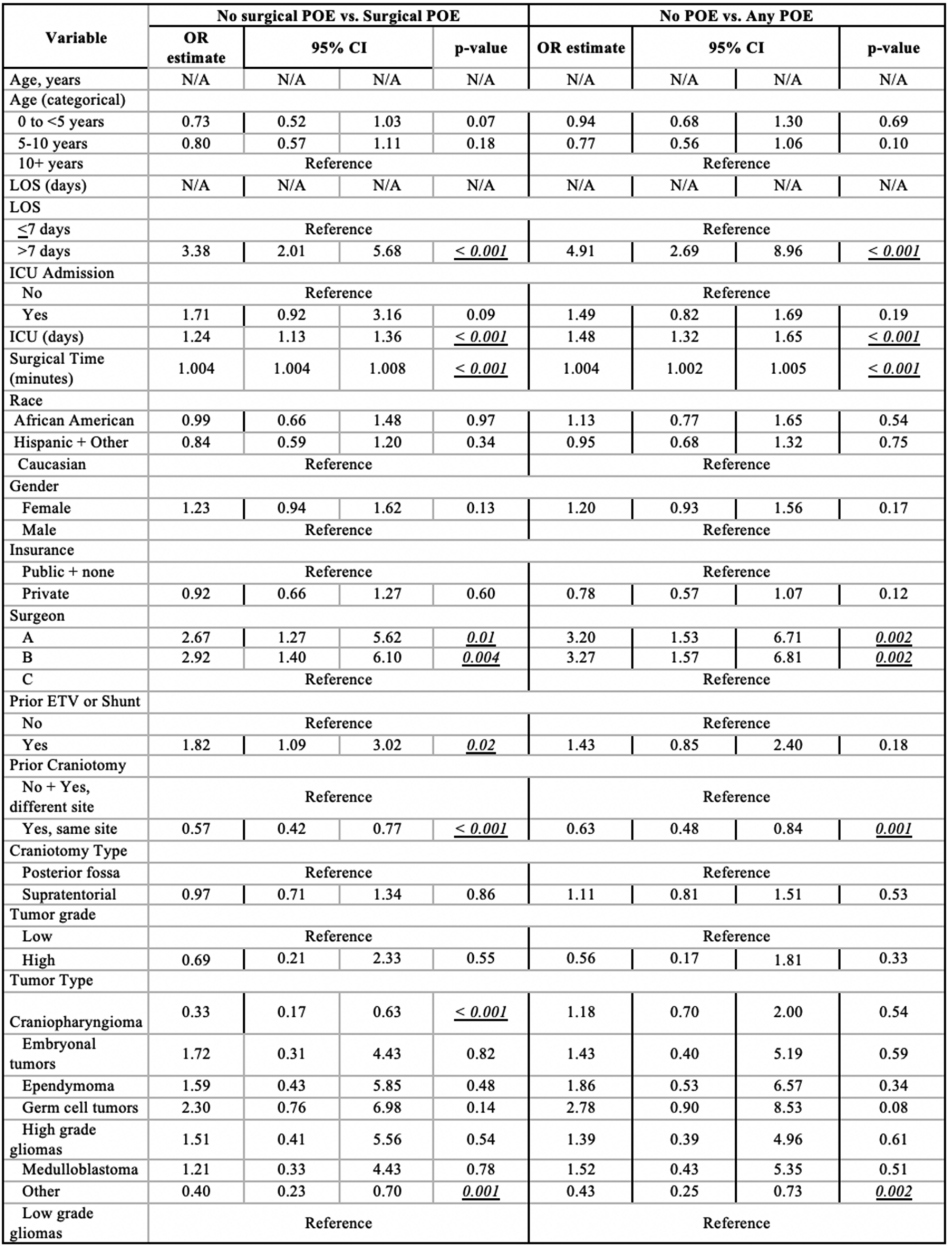
Multivariate analysis utilized to determine association between each variable and occurrence of surgical POE / any POE

### Any POE

Bivariate analysis of the occurrence of any POE revealed several significant variables (Online Resource 4): continuous age (OR 0.98, *p* = 0.03), age 0 to < 5 years (OR 1.33, *p* = 0.03), continuous LOS (OR 1.78, *p* < 0.001), LOS > 7 days (OR 24.36, *p* < 0.001), ICU admission (OR 5.31, *p* < 0.001), ICUS (OR 1.82, *p* < 0.001), surgical time (OR 1.006, *p* < 0.001), surgeon A (OR 1.82, *p* = 0.02), surgeon B (OR 2.02, *p* = 0.007), prior ETV or shunt (OR 2.06, *p* < 0.001), craniopharyngioma (OR 1.57, *p* = 0.03), and germ cell tumor (OR 2.33, *p* = 0.04). In that same analysis, several variables were significantly less likely to result in any POE, including private insurance (OR 0.78, *p* = 0.04), prior craniotomy at the same site (OR 0.61, *p* < 0.001), supratentorial craniotomy (OR 0.80, *p* = 0.04), high grade glioma (OR 0.59, *p* = 0.01), and other tumor type (OR 0.43, *p* < 0.001). Multivariate analysis showed the following to be significant predictors of any POE (Table 4): LOS > 7 days (OR 4.91, *p* < 0.001), ICUS (OR 1.48, *p* < 0.001), surgical time (OR 1.004, *p* < 0.001), surgeon A (OR 3.20, *p* = 0.002), and surgeon B (OR 3.27, *p* = 0.002). Prior craniotomy at the same site (OR 0.63, *p* = 0.001) and other tumor type (OR 0.43, *p* = 0.002) were less likely to result in any POE.

## Discussion

This study represents the largest and most comprehensive effort to evaluate POEs following elective craniotomy for tumor in children. We identified a number of variables, many of which were positively associated (i.e., increased odds), with the occurrence of one or more POE within the first 90 days of the index operation. We will discuss our findings and their implications.

For both surgical only and all POEs, we found extended LOS (>7 days), ICUS, surgical time, and attending surgeon as being positive predictors; repeat craniotomy at the same site was protective (i.e., lowered odds). It is understandable how LOS and ICU are associated with POE as any POE could easily drive both. In our previous study, we evaluated the inverse relationship – whether POE was predictive of extended LOS – and found a 30-fold increase in the odds [17]. In a study by Malhotra et al., major complications following primary ependymoma resection—such as persistent cranial nerve deficits and hematoma or infection requiring reoperation—prolonged LOS by 22 days on average [13]. Another group demonstrated that a longer ICU stay increased the risk of catheter-associated meningitis in children with brain tumors [16].

While the odds ratio for surgical time was barely above 1.0 (OR 1.004), the unit of measurement was minutes (min). In other words, for every 1 additional minute of operative time, the odds of a POE increase by 0.4%. Like LOS and ICUS, surgical time and POE represent a correlation, not causation. A longer operation by itself does not directly cause POEs but rather is reflective of the complexity of the operation. In the pediatric literature, longer duration of surgery has been shown to be a risk factor for unplanned reintubation [19], surgical site infection [20, 21], and other POEs [20]. We, too, found it to be an independent driver of 90-day readmission [18]. In the adult population, Anthofer et al. reported that increased operative time for craniotomy was predictive of medical POEs generally; it remained a significant risk factor in the multivariate analyses of venous thromboembolism (VTE) and sepsis [22]. Another study of adults undergoing elective craniotomy for meningioma showed that operative time *≥* 310 min was a significant risk factor for postoperative VTE [23].

Finally, the attending surgeon of record was an independent positive predictor of POE. This is because at our institution, most tumors, particularly those that were of high complexity, were performed by two surgeons (A & B) whereas the other surgeons (grouped as “C”) did more straightforward cases. Therefore, it comes as no surprise that patients of surgeons A & B would have more POEs than surgeon C. So, while Steinbok et al. found that surgeons who did most of the intracranial tumor cases (and had more years of experience) had lower rates of neurological deficits compared to surgeons who did less [10], the true comparison requires controlling for case mix complexity rather than simply looking at overall volume.

A fascinating discovery was that redo surgery (i.e., prior craniotomy at the same site) was protective against POEs. One could easily postulate that redo surgery is more challenging because of changes brought about after the prior operation(s) and treatments. The literature, however, is mixed. In a study of adult patients, Anthofer et al. found that prior craniotomy was a significant protective factor against medical POEs after elective craniotomy [22], while another group demonstrated that redo craniotomy for high-grade gliomas increased the odds of postoperative systemic infection and worsened neurological status [24]. Lassen et al. reported that prior craniotomy was not a statistically significant risk factor for CSF leak after tumor surgery in children [25]. In our population, a patient with prior craniotomy at the same site is usually undergoing redo surgery for residual or recurrent tumor; thus, the greatest danger to surrounding structures and risk for injury was highest in the first surgery. Second, tumor volume is smaller in patients undergoing surgery for residual or recurrent disease. Still, the impact of prior surgery is not as simple as we have defined it but likely has more to do with other factors such as the tumor location, tumor type, tumor consistency, number of prior operations and the goal(s) of surgery.

There were additional factors associated with surgical-only POEs. Previously treated hydrocephalus increased the odds of a surgical POE by 82%. There is literature to suggest that hydrocephalus, whether treated or untreated, prior to a craniotomy poses a greater risk of post-operative complications [21, 25, 26]. In one pediatric study, untreated preoperative hydrocephalus was a significant risk factor for new or worsening neurological deficit, as well as CSF leak, after craniotomy or open biopsy for tumor [25]. The same variable was found to be significant in our previous analysis of extended LOS [17]. As with that study, we hypothesize that hydrocephalus adds to the dimensionality of the patient, elevating the risk of that child having a POE.

Finally, the variable “tumor type” yielded some interesting results. When compared to the low grade glioma category, craniopharyngioma had 3 times lower odds of a POE, respectively. There may be several reasons for this. First, postoperative sodium disturbances and other endocrinopathies, which we classified as a medical POE for the purpose of this study (admittedly a valid criticism), are well described with craniopharyngioma surgery [27–29]. Second, for many years, we have advocated for less aggressive surgery for this particular tumor. The majority of craniopharyngioma patients seen at our institution already come with endocrinopathies from their prior surgery; operations that we do are primarily very limited resections in order to optimize the child’s tumor burden in preparation for proton beam therapy.

### Strengths and limitations

The strengths of this study lie in the volume of cases examined, the diversity of diagnoses and outcomes, and the long study time period. The data was collected in a prospective manner by a single person with extensive clinical knowledge of pediatric neurosurgery. The database was created with the goal of capturing as much relevant information as possible, but some variables (e.g., tumor volume, intraoperative blood loss) were not included, and others may have been too broad in scope. This was a broad exploratory analysis, rather than a more focused explanatory analysis. Future studies would probably be of greater value if variables were limited, with more precise definitions and incorporating information that is more reflective of the current state of pediatric neurosurgical oncology. Additionally, this being a single-center study based on the outcomes of three surgeons reduces the generalizability of our findings. Expanding this study to include other institutions would help to validate our conclusions. Finally, there is an inherent element of subjectivity and therefore potential bias when evaluating postoperative complications. This highlights the need to develop a standardized set of POEs (definitions and grades) that can be adopted for future publications.

## Conclusion

This is the largest study that evaluates 90-day POEs following an elective tumor removal in the pediatric population. POEs are surgically driven; medical ones have little impact in this population. This study strongly suggests that all patients who undergo a craniotomy for tumor removal should be assessed for risk and any measures to mitigate those risks should be undertaken.

## Supplementary Information

Below is the link to the electronic supplementary material.

Supplementary Material 1

## Data Availability

No datasets were generated or analysed during the current study.

## Abbreviations

LOS: Length of stay
POE: Postoperative event
CSF: Cerebrospinal fluid
VTE: Venous thromboembolism
DI: Diabetes insipidus

## References

[1] Ostrom QT, Cioffi G, Gittleman H et al (2019) CBTRUS statistical report: primary brain and other central nervous system tumors diagnosed in the united States in 2012–2016. Neuro-Oncol 21(Supplement5):v1–v100. 10.1093/neuonc/noz150 31675094 10.1093/neuonc/noz150 PMC6823730

[2] Albright AL, Pollack IF, Adelson PD, Solot JJ (1999) Outcome data and analysis in pediatric neurosurgery. Neurosurgery. 1:101 10.1097/00006123-199907000-00025 10414572

[3] Neervoort FW, Van Ouwerkerk WJR, Folkersma H, Kaspers GJL, Vandertop WP (2010) Surgical morbidity and mortality of pediatric brain tumors: a single center audit. Childs Nerv Syst 26(11):1583–1592. 10.1007/s00381-010-1086-1 20204381 10.1007/s00381-010-1086-1 PMC2974195

[4] Foster MT, Hennigan D, Grayston R et al (2021) Reporting morbidity associated with pediatric brain tumor surgery: are the available scoring systems sufficient? J Neurosurg Pediatr 27(5):556–565. 10.3171/2020.9.PEDS20556 33636703 10.3171/2020.9.PEDS20556

[5] Campbell E, Beez T, Todd L (2017) Prospective review of 30-day morbidity and mortality in a paediatric neurosurgical unit. Childs Nerv Syst 33(3):483–489. 10.1007/s00381-017-3358-5 28247111 10.1007/s00381-017-3358-5 PMC5368193

[6] Von Lehe M, Kim HJ, Schramm J, Simon M (2013) A comprehensive analysis of early outcomes and complication rates after 769 craniotomies in pediatric patients. Childs Nerv Syst 29(5):781–790. 10.1007/s00381-012-2006-3 23274639 10.1007/s00381-012-2006-3

[7] Drake JM, Riva-Cambrin J, Jea A, Auguste K, Tamber M, Lamberti-Pasculli M (2010) Prospective surveillance of complications in a pediatric neurosurgery unit: clinical Article. J Neurosurg Pediatr 5(6):544–548. 10.3171/2010.1.PEDS09305 20515324 10.3171/2010.1.PEDS09305

[8] Van Lindert EJ, Delye H, Leonardo J (2014) Prospective review of a single center’s general pediatric neurosurgical intraoperative and postoperative complication rates: clinical Article. J Neurosurg Pediatr 13(1):107–113. 10.3171/2013.9.PEDS13222 24236448 10.3171/2013.9.PEDS13222

[9] Ajmera S, Motiwala M, Lingo R et al (2019) Emergent and urgent craniotomies in pediatric patients: resource utilization and cost analysis. Pediatr Neurosurg 54(5):301–309. 10.1159/000501042 31401624 10.1159/000501042

[10] Steinbok P, Mangat JS, Kerr JM et al (2013) Neurological morbidity of surgical resection of pediatric cerebellar Astrocytomas. Childs Nerv Syst 29(8):1269–1275. 10.1007/s00381-013-2171-z 23715810 10.1007/s00381-013-2171-z

[11] Miao Y, Fan K, Peng X et al (2023) Postoperative hypothalamic-pituitary dysfunction and long-term hormone replacement in patients with childhood-onset craniopharyngioma. Front Endocrinol 14:1241145. 10.3389/fendo.2023.1241145 10.3389/fendo.2023.1241145 PMC10657986 38027203

[12] Goodden J, Pizer B, Pettorini B et al (2014) The role of surgery in optic pathway/hypothalamic gliomas in children: clinical Article. J Neurosurg Pediatr 13(1):1–12. 10.3171/2013.8.PEDS12546 24138145 10.3171/2013.8.PEDS12546

[13] Malhotra AK, Nobre L, Ibrahim GM et al (2024) Complications following resection of primary and recurrent pediatric posterior fossa ependymoma. J Neurosurg Pediatr 33(4):367–373. 10.3171/2023.11.PEDS23364 38241689 10.3171/2023.11.PEDS23364

[14] Williams C, Simon TD, Riva-Cambrin J, Bratton SL (2012) Hyponatremia with intracranial malignant tumor resection in children: clinical Article. J Neurosurg Pediatr 9(5):524–529. 10.3171/2012.1.PEDS11465 22546031 10.3171/2012.1.PEDS11465 PMC3358130

[15] Bokhari R, Elkaim LM, Shlobin NA et al (2023) Vasospasm following brain tumor resection in children: institutional experience and systematic review. J Neurosurg Pediatr 32(3):343–350. 10.3171/2023.3.PEDS22373 37327188 10.3171/2023.3.PEDS22373

[16] Chihi M, Gembruch O, Ahmadipour Y et al (2020) Predictors of catheter-associated meningitis in pediatric patients after brain tumor surgery: A 10-year single center experience. J Neurol Sci 418:117100. 10.1016/j.jns.2020.117100 32861083 10.1016/j.jns.2020.117100

[17] Lesha E, Roach JT, Miller LE et al (2025) Length of stay following elective craniotomy for tumor resection in children and young adults: a retrospective case series. J Neurooncol 171(3):651–658. 10.1007/s11060-024-04887-w 39612129 10.1007/s11060-024-04887-w PMC11729059

[18] Lesha E, Laird DG, Nichols CS et al (2025) Variables associated with 90-day readmission following craniotomy for tumor in the pediatric population. J Neurooncol 173(3):759–767. 10.1007/s11060-025-05021-0 40232620 10.1007/s11060-025-05021-0 PMC12170774

[19] Drapeau AI, Mpody C, Gross MA, Lemus R, Tobias JD, Nafiu O (2024) Factors associated with unplanned Post-Craniotomy Re-intubation in children: A NSQIP-Pediatric^®^ analysis. J Neurosurg Anesthesiol 36(1):37–44. 10.1097/ANA.0000000000000871 36136605 10.1097/ANA.0000000000000871

[20] Iglesias NJ, Arrowood R, Montgomery L, Leeper E, Tsao KJ, Iglesias JL (2022) Operative time is independently associated with morbidity in pediatric complicated appendicitis. J Surg Res 276:143–150. 10.1016/j.jss.2022.02.045 35339782 10.1016/j.jss.2022.02.045

[21] Sáenz A, Badaloni E, Grijalba M, Villalonga JF, Argañaraz R, Mantese B (2021) Risk factors for surgical site infection in pediatric posterior fossa tumors. Childs Nerv Syst 37(10):3049–3056. 10.1007/s00381-021-05256-y 34142227 10.1007/s00381-021-05256-y

[22] Anthofer J, Wester M, Zeman F, Brawanski A, Schebesch KM (2016) Case-Control study of patients at risk of medical complications after elective craniotomy. World Neurosurg 91:58–65. 10.1016/j.wneu.2016.03.087 27062920 10.1016/j.wneu.2016.03.087

[23] Nunno A, Li Y, Pieters TA et al (2019) Risk factors and associated complications of symptomatic venous thromboembolism in patients with craniotomy for meningioma. World Neurosurg 122:e1505–e1510. 10.1016/j.wneu.2018.11.091 30468929 10.1016/j.wneu.2018.11.091

[24] Chang SM, Parney IF, Mcdermott M et al (2003) Perioperative complications and neurological outcomes of first and second craniotomies among patients enrolled in the glioma outcome project. J Neurosurg 98(6):1175–1181. 10.3171/jns.2003.98.6.1175 12816260 10.3171/jns.2003.98.6.1175

[25] Lassen B, Helseth E, Egge A, Due-Tønnessen BJ, Rønning P, Meling TR (2012) Surgical mortality and selected complications in 273 consecutive craniotomies for intracranial tumors in pediatric patients. Neurosurgery 70(4):936–943. 10.1227/NEU.0b013e31823bcc61 21993188 10.1227/NEU.0b013e31823bcc61

[26] Tabakow P, Weiser A, Burzynska M, Blauciak P (2022) Endoscopic third ventriculostomy before surgery of third ventricle and posterior fossa tumours decreases the risk of secondary hydrocephalus and early postoperative complications. Neurosurg Rev 45(1):771–781. 10.1007/s10143-021-01570-w 34291350 10.1007/s10143-021-01570-w PMC8827142

[27] Ortiz Torres M, Shafiq I, Mesfin FB (2025) Pediatric craniopharyngioma. StatPearls [Internet]. StatPearls Publishing. https://www.ncbi.nlm.nih.gov/books/NBK519027/

[28] Puget S, Garnett M, Wray A et al (2007) Pediatric craniopharyngiomas: classification and treatment according to the degree of hypothalamic involvement. J Neurosurg Pediatr 106(1):3–12. 10.3171/ped.2007.106.1.3 10.3171/ped.2007.106.1.3 17233305

[29] Jeswani S, Nuño M, Wu A et al (2016) Comparative analysis of outcomes following craniotomy and expanded endoscopic endonasal transsphenoidal resection of craniopharyngioma and related tumors: a single-institution study. J Neurosurg 124(3):627–638. 10.3171/2015.3.JNS142254 26361276 10.3171/2015.3.JNS142254

