# Supplementary material for: Post-operative events following elective craniotomy for tumor in children
Source: J Neurooncol. 2025 Oct 20;176(1):28. doi: 10.1007/s11060-025-05239-y (PMC12537591; doi:10.1007/s11060-025-05239-y)
Supplement: Supplementary file 1 — Supplementary Material 1 [file 11060_2025_5239_MOESM1_ESM.docx]

**Supplemental Material**

**Online Resource 1**: Frequency of Surgical POEs


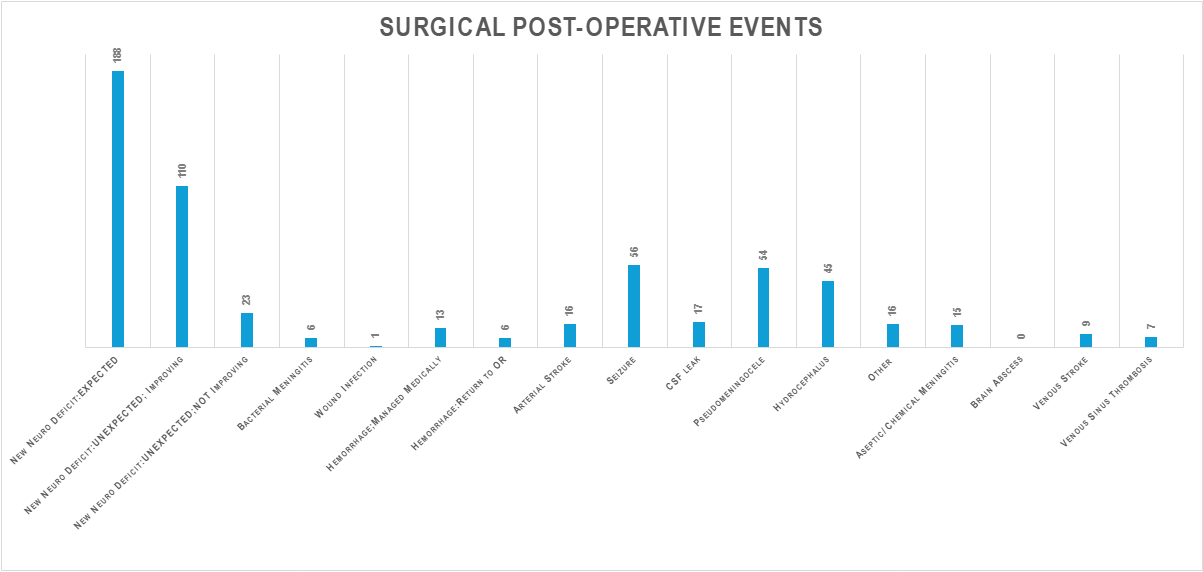


**Online Resource 2:** Frequency of Medical POEs


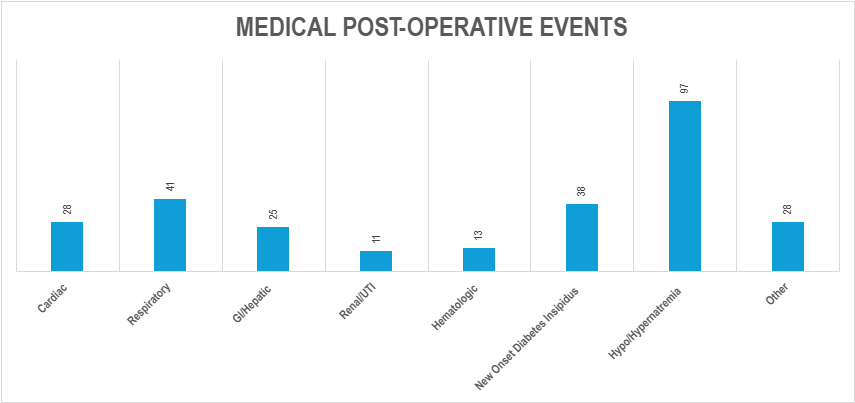


**Online Resource 3:** Bivariate analysis results utilized to determine association between each variable and occurrence of surgical POE

| **Variable** |  | **Bivariate** | | | |
| --- | --- | --- | --- | --- | --- |
|  |  | **OR estimate** | **95% CI** | | **p-value** |
| Age, years |  | 0.99 | 0.97 | 1.01 | 0.19 |
| Age (categorical) |  |  | | | |
| 0 to <5 years |  | 1.16 | 0.89 | 1.52 | 0.28 |
| 5-10 years |  | 0.99 | 0.75 | 1.31 | 0.96 |
| 10+ years |  | Reference | | | |
| LOS (days) |  | 1.51 | 1.43 | 1.60 | *< 0.001* |
| LOS |  |  | | | |
| <7 days |  | Reference | | | |
| >7 days |  | 12.17 | 8.39 | 17.65 | *< 0.001* |
| ICU Admission |  |  | | | |
| No |  | Reference | | | |
| Yes |  | 4.57 | 2.65 | 7.86 | *< 0.001* |
| ICU (days) |  | 1.48 | 1.39 | 1.58 | *< 0.001* |
| Surgical Time (minutes) |  | 1.006 | 1.005 | 1.007 | *< 0.001* |
| Race |  |  | | | |
| African American |  | 0.95 | 0.68 | 1.32 | 0.74 |
| Hispanic + Others |  | 0.86 | 0.64 | 1.15 | 0.31 |
| Caucasian |  | Reference | | | |
| Gender |  |  | | | |
| Female |  | 1.12 | 0.89 | 1.40 | 0.34 |
| Male |  | Reference | | | |
| Insurance |  |  | | | |
| Public or none |  | Reference | | | |
| Private |  | 0.84 | 0.65 | 1.09 | 0.20 |
| Surgeon |  |  | | | |
| A |  | 1.51 | 0.88 | 2.59 | 0.14 |
| B |  | 1.81 | 1.06 | 3.11 | *0.03* |
| C |  | Reference | | | |
| Prior ETV or Shunt |  |  | | | |
| No |  | Reference | | | |
| Yes |  | 2.58 | 1.69 | 3.93 | *< 0.001* |
| Prior Craniotomy |  |  | | | |
| No + Yes, different site |  | Reference | | | |
| Yes, same site |  | 0.57 | 0.45 | 0.73 | *< 0.001* |
| Craniotomy Type |  |  | | | |
| Posterior fossa |  | Reference | | | |
| Supratentorial |  | 0.70 | 0.55 | 0.88 | *0.002* |
| Tumor grade |  |  | | | |
| Low |  | Reference | | | |
| High |  | 0.99 | 0.79 | 1.24 | 0.94 |
| Tumor Type |  |  | | | |
| Craniopharyngioma |  | 0.56 | 0.35 | 0.92 | *0.02* |
| Embryonal tumors |  | 1.06 | 0.70 | 1.59 | 0.79 |
| Ependymoma |  | 0.90 | 0.63 | 1.29 | 0.57 |
| Germ cell tumors |  | 2.25 | 1.04 | 4.90 | *0.04* |
| High grade gliomas |  | 0.72 | 0.47 | 1.09 | 0.12 |
| Medulloblastoma |  | 0.77 | 0.53 | 1.10 | 0.15 |
| Other |  | 0.42 | 0.27 | 0.65 | *< 0.001* |
| Low grade gliomas |  | Reference | | | |
| Complications |  |  | | | |
| No |  | Reference | | | |
| Yes |  | 46.47 | 14.40 | 149.98 | *< 0.001* |

**Online Resource 4:** Bivariate analysis results utilized to determine association between each variable and occurrence of any POE

| **Variable** |  | **Bivariate** | | | |
| --- | --- | --- | --- | --- | --- |
|  |  | **OR estimate** | **95% CI** | | **p-value** |
| Age, years |  | 0.98 | 0.96 | 0.99 | *0.03* |
| Age (categorical) |  |  | | | |
| 0 to <5 years |  | 1.33 | 1.04 | 1.72 | *0.03* |
| 5-10 years |  | 1.02 | 0.78 | 1.32 | 0.91 |
| 10+ years |  | Reference | | | |
| LOS (days) |  | 1.78 | 1.66 | 1.91 | < 0.001 |
| LOS |  |  | | | |
| <7 days |  | Reference | | | |
| >7 days |  | 24.36 | 14.57 | 40.71 | *< 0.001* |
| ICU Admission |  |  | | | |
| No |  | Reference | | | |
| Yes |  | 5.31 | 3.21 | 8.76 | *< 0.001* |
| ICU (days) |  | 1.82 | 1.67 | 1.99 | *< 0.001* |
| Surgical Time (minutes) |  | 1.006 | 1.005 | 1.007 | *< 0.001* |
| Race |  |  | | | |
| African American |  | 1.14 | 0.84 | 1.55 | 0.40 |
| Hispanic + Others |  | 1.06 | 0.81 | 1.39 | 0.66 |
| Caucasian |  | Reference | | | |
| Gender |  |  | | | |
| Female |  | 1.11 | 0.90 | 1.38 | 0.32 |
| Male |  | Reference | | | |
| Insurance |  |  | | | |
| Public or none |  | Reference | | | |
| Private |  | 0.78 | 0.61 | 0.99 | *0.04* |
| Surgeon |  |  | | | |
| A |  | 1.82 | 1.09 | 3.04 | *0.02* |
| B |  | 2.02 | 1.21 | 3.37 | *0.007* |
| C |  | Reference | | | |
| Prior ETV or Shunt |  |  | | | |
| No |  | Reference | | | |
| Yes |  | 2.06 | 1.35 | 3.13 | *< 0.001* |
| Prior Craniotomy |  |  | | | |
| No + Yes, different site |  | Reference | | | |
| Yes, same site |  | 0.61 | 0.49 | 0.76 | *< 0.001* |
| Craniotomy Type |  |  | | | |
| Posterior fossa |  | Reference | | | |
| Supratentorial |  | 0.80 | 0.64 | 0.99 | *0.04* |
| Tumor grade |  |  | | | |
| Low |  | Reference | | | |
| High |  | 0.84 | 0.68 | 1.03 | 0.10 |
| Tumor Type |  |  | | | |
| Craniopharyngioma |  | 1.57 | 1.05 | 2.37 | *0.03* |
| Embryonal tumors |  | 1.09 | 0.74 | 1.61 | 0.67 |
| Ependymoma |  | 0.91 | 0.65 | 1.28 | 0.58 |
| Germ cell tumors |  | 2.33 | 1.06 | 5.13 | *0.04* |
| High grade gliomas |  | 0.59 | 0.39 | 0.89 | *0.01* |
| Medulloblastoma |  | 0.80 | 0.57 | 1.13 | 0.21 |
| Other |  | 0.43 | 0.29 | 0.64 | *< 0.001* |
| Low grade gliomas |  | Reference | | | |
| Complications |  |  | | | |
| No |  | Reference | | | |
| Yes |  | 101.89 | 14.04 | 739.55 | *< 0.001* |
